# A comparison approach to explain risks related to x-ray imaging for scoliosis

**DOI:** 10.1186/1748-7161-8-S1-O37

**Published:** 2013-06-03

**Authors:** N Pace, L Ricci

**Affiliations:** 1 BIOtech - Interdepartmental Center on Biomedical Technologies, Università di Trento, Trento, Italy; 2 Dipartimento di Fisica,Università di Trento, Trento, Italy

## Background

X-ray imaging is frequently used as a diagnostic approach for scoliosis in children and adolescents. X-ray procedures are justified only when expected benefits exceed related risks. While benefits are well known to physicians, radiological risk awareness can be vague, impeding an optimal communication with patients' parents, and possibly leading to discomfort and anxiety.

## Aim

To suggest a risk comparison approach for better communicating the radiological risks related to X-ray investigation of scoliosis.

## Methods

Starting point of the analysis is the Linear Non Threshold (LNT) assumption for radiation stochastic effect [1]: for effective doses (E) below 100 mSv, the probability of future stochastic damage is linearly related to E. This allows to add E from different sources to calculate a cumulative risk of health detriment. Data coming from literature were gathered to determine the average E delivered during X-ray investigation of scoliosis. Subsequently, the major natural sources of radiation, namely cosmic rays, 40K, 222Rn and other radionuclides, were considered. The average E due to these natural sources was compared with E due to the imaging of the vertebral column.

## Results

For a single standard scoliosis radiographic examination [2], E ranges from 0.2 to 0.35 mSv [3]. Therefore, the LNT assumption can be used. The main natural radiation source is 222Rn, which on average accounts for 1.2 mSv / y (range 0.2 +/-10 mSv / y). Cosmic rays (0.4 mSv / y, range 0.3 +/-1.0) and terrestrial gamma rays from other radionuclides (0.8 mSv / y, range 0.3 +/- 1.4) are additional sources of natural radiation. Moreover, flying from Europe to North America accounts for 0.03-0.05 mSv, and because of the unavoidable presence of 40K, consuming 1,000 bananas, or carrots, accounts for approx. 0.1 mSv. Overall, 65% of the world population is expected to be exposed to an annual E between 1 and 3 mSv (average 2.4 mSv) [1].

## Conclusions

Data coming from literature show how, on average, the effective annual dose coming from natural sources greatly exceeds the effective dose due to x-ray imaging for scoliosis. This information can play a key role in the relationship between physicians and patients.
